# Differential Responses of Emergent Intertidal Coral Reef Fauna to a Large-Scale El-Niño Southern Oscillation Event: Sponge and Coral Resilience

**DOI:** 10.1371/journal.pone.0093209

**Published:** 2014-03-27

**Authors:** Francisco Kelmo, James J. Bell, Simone Souza Moraes, Rilza da Costa Tourinho Gomes, Eduardo Mariano-Neto, Martin J. Attrill

**Affiliations:** 1 Instituto de Biologia, Universidade Federal da Bahia, Campus Universitário de Ondina, Salvador, Bahia, Brazil; 2 School of Biological Sciences, Victoria University of Wellington, Wellington, New Zealand; 3 Instituto de Geociências, Universidade Federal da Bahia, Campus Universitário de Ondina, Salvador, Bahia, Brazil; 4 Curso de Pós-Graduação em Desenvolvimento Humano e Responsabilidade Social, Fundação Visconde de Cairu, Rua do Salete, 50, Barris, Salvador, Bahia, Brazil; 5 Marine Institute, Plymouth University, Drake Circus, Plymouth, Devon, United Kingdom; Seagrass Ecosystem Research Group, Swansea University, United Kingdom

## Abstract

There is a paucity of information on the impacts of the 1997–8 El Niño event and subsequent climatic episodes on emergent intertidal coral reef assemblages. Given the environmental variability intertidal reefs experience, such reefs may potentially be more resilient to climatic events and provide important insights into the adaptation of reef fauna to future ocean warming. Here we report the results of a 17-year (1995–2011) biodiversity survey of four emergent coral reef ecosystems in Bahia, Brazil, to assess the impact of a major El Niño event on the reef fauna, and determine any subsequent recovery. The densities of two species of coral, *Favia gravida* and *Siderastrea stellata*, did not vary significantly across the survey period, indicating a high degree of tolerance to the El Niño associated stress. However, there were marked decreases in the diversity of other taxa. Molluscs, bryozoans and ascidians suffered severe declines in diversity and abundance and had not recovered to pre-El Niño levels by the end of the study. Echinoderms were reduced to a single species in 1999, *Echinometra lucunter*, although diversity levels had recovered by 2002. Sponge assemblages were not impacted by the 1997–8 event and their densities had increased by the study end. Multivariate analysis indicated that a stable invertebrate community had re-established on the reefs after the El Niño event, but it has a different overall composition to the pre-El Niño community. It is unclear if community recovery will continue given more time, but our study highlights that any increase in the frequency of large-scale climatic events to more than one a decade is likely to result in a persistent lower-diversity state. Our results also suggest some coral and sponge species are particularly resilient to the El Niño-associated stress and therefore represent suitable models to investigate temperature adaptation in reef organisms.

## Introduction

Coral reefs around the world have already been degraded as a result human impacts from many local-scale and global scale impacts [1], [2], [3], and there is now increasing interest in the resilience of reef species to such stressors [4]. Of particular concern are global-scale impacts such as ocean acidification, sea surface temperature increase and the frequency of large-scale climatic events (e.g. the El-Niño Southern Oscillation – ENSO). There has been a recent focus on those organisms inhabiting marginal habitats as they often survive under sub-optimal conditions close to their physiological limits, and may therefore be adapted to higher levels of physiological stress [5]. Organisms inhabiting such environments have the potential to serve as models for understanding the impacts of global climate change and large-scale climatic events.

Emergent intertidal coral reefs are common around the world [5], and organisms inhabiting these environments will be subject to much larger fluctuations in temperature and solar radiation stress compared to nearby subtidal organisms [6]. While this may mean such communities are more resilient to climatic related impacts than subtidal organisms, the reverse may actually be true, communities may be less resilient to stressors as they are living close to their physiological tolerance limits.

The effects of large-scale El-Niño Southern Oscillation (hereafter ENSO) events on coral communities have been well described, particularly as a result of the 1997–8 event that had devastating impacts on many coral reefs (e.g. [7], [8]). However, the influences of these large-scale events on other dominant reef organisms are less well known. Such ENSO anomalies are normally accompanied by reduced nutrient replenishment to surface waters [9], with subsequent declines in phytoplankton production [10], [11] and the disruption of trophic links between higher level consumers [12]; this has the potential to result in irreversible damage to the coral reef-associated organisms and result in changes to overall community structure.

During the 1997–8 ENSO period, the northeastern coast of Brazil experienced sustained surface warming, causing prolonged increased sea surface temperatures [13]. These environmental changes had major impacts on the coral reef communities over large spatial scales [14]. In northern Bahia seawater temperature increased about 1°C above its previous maximum and, concurrently, there was reduced rainfall causing a decrease in the amount of continental sediment transported to the sea from the local river discharge. This resulted in reduced turbidity and subsequently increased penetration of solar radiation, accompanied by the prolonged warming of shallow waters. The impact of solar radiation was accentuated further as a result of significantly reduced cloud cover during the ENSO event, resulting in high levels of ultra-violet radiation (UVR) potentially reaching the reef community. Previous studies (e.g. [14], [15], [16], [17]) of northern Bahian subtidal reefs have described widespread coral bleaching and mortality of reef-associated invertebrates following the 1997–8 ENSO event, which was associated with observed increases in seawater temperature. These declines were considered most likely a result of the combined and synergistic effects of elevated seawater temperatures and changes in irradiance, sedimentation and calm sea conditions that reduced wind driven water flow patterns [18], [19], [20], [21].

To provide a contrast to patterns observed in subtidal reef systems, here we report the results of a 17-year study of the coastal emergent reefs of Northern Bahia, and provide an overview of the impacts of the 1997–8 ENSO on the intertidal reef-associated fauna and their subsequent recovery trajectories. This habitat is unusual in that coral species, and their associated community, survive in a fully intertidal reef top environment and therefore experience much greater variability in environmental conditions compared with the local subtidal reef ecosystem. We hypothesise, therefore, that the species here will be more tolerant to fluctuations in environmental conditions and therefore the community will show a less dramatic response to ENSO events than witnessed in adjacent subtidal coral reef assemblages.

## Methods

### Study area

This study focused on four coastal emergent reefs in Bahia, Brazil [Abaí (12°40′37″S/38°05′23W), Guarajuba (12°39′00″S/38°03′43″W), Itacimirim (12°38′13″S/38°02′51″W) and Praia do Forte (12°34′42″S/37°58′59″W)]. These reefs run parallel to the coastline and their dimensions vary from 20 m to 500 m wide [22]. They occur in the fore reef zone in waters less than 14 m deep, and their back zones usually slope downward into the beach, which is comprised of quartz-sands. They have horizontal tops that are uncovered during low tide, the sample habitat for this study, their exposed surfaces, eroded due to Holocene sea-level fluctuations, have irregular thin columnar structures, cavities, meandering channels, and small caves, where small heads of living coral andothers reef invertebrates exist along with green, red and brown algae. These reefs are located on the narrowest part of the Eastern Brazilian Continental Shelf (average width 15 km between the Sao Francisco and Doce Rivers) and extend 20 km between the beaches of Abaí and Praia do Forte (see Fig. 1 in [23]). The tidal regime is semi-diurnal. The data available for tidal ranges are from the Port of Salvador, the average range at spring tide is 2.4 m and at neap tide 0.1 m. The tidal currents are fairly consistent, though influenced by the strength and direction of the wind; the average current velocity is approximately 1.5 ms^−1^. For a full description of the geological history and morphology of the reefs see [24].

The coastal belt of the State of Bahia has a tropical humid climate. Annual average rainfall ranges between 1,300 mm in the north of the study area to 1,900 mm around Salvador City to the south, with no marked seasonal rainfall pattern. Average daily air temperatures range from 23°C (winter) to 28°C (summer), with mean daily sea-surface temperatures ranging from 25°C (winter) to 28°C (summer); maximum SST occurs between December and February. Annual average salinity is relatively constant (35–36 ppt), although within emergent reef-top shallow pools, salinity can range from 35 to 39 ppt (see [6]). The pH of seawater varies between 8.1 and 8.2, with no clear seasonal patterns (see [15], [23]). The coast is influenced by winds arising from the NE and E during the spring-summer, and winds coming from the SE and E during the autumn-winter season. Moreover, during the autumn-winter period, the winds coming from the SSE that are associated with the periodic advance of the Atlantic Polar Front, reinforce the trade winds from the SE [25]. This pattern of wind circulation is disrupted by the quasi-cyclic environmental phenomenon known as the El Niño/La Niña, combined as the El Niño Southern Oscillation, with several major climatic perturbations having been recorded in recent times [13], [26].

### Environmental data

Large-scale environmental parameters for the survey area (sea surface temperature, solar irradiance, air temperature, rainfall, and cloud cover) were obtained from the Brazilian Meteorological Institute [INMET (http://www.inmet.gov.br/portal/index.php?r=home/page&page=rede_estacoes_conv_graf)]. INMET data are collected three times a day and the values presented in this paper represent the annual average of these data. Local physicochemical data (seawater temperature, salinity, pH, and turbidity) were recorded at all four reefs (10 replicates/reef giving 40 measurements spread over the sampling period). Temperature, salinity, and pH were recorded using a YSI63 (Yellow Spring Industries) electronic field meter. Turbidity at high water was assessed using a Secchi disk that was deployed from a boat for coral reef walls (CRW) and shallow bank reefs (SBR) environments. From 2001, we recorded turbidity and other local data using a Multiparameter Water Quality Meter (U5210); however, based on the similarity in the results obtained from the different methods we present the same type of measurement throughout the years to ensure consistency (see Table S1 in [15]).

### Sampling and identification

Density data on the associated invertebrate community (Porifera, Cnidaria, Mollusca, Bryozoa, Echinodermata and Ascidiacea) from the reef tops of the four different coastal emergent reefs (Praia do Forte, Itacimirim, Guarajuba and Abaí) were collected annually (between April and May) from 1995 to 2011. Density estimates were taken within 35 1 m^2^ quadrats positioned haphazardly on each reef, giving a total of 140 quadrats per year and 2,380 quadrats in total over the survey period. During the first two years of this investigation, samples of each species were brought to the laboratory to confirm identity. Additional samples were collected in subsequent years for taxonomical purposes when necessary. The number of bleached coral colonies was also recorded. A permanent license to collect zoological material (N° 37409-1) was provided by the Ministry of the Environment, Chico Mendes Institute of Biodiversity Conservation, Authorisation System and Information on Biodiversity (Normative Instruction N° 154/2007). Through the authentication code N° 78456982, any citizen can check the authenticity or legality of this document, by examining the Sisbio/ICMBio information on the Internet (www.icmbio.gov.br/sisbio). No other specific permissions were required as this was an entirely field-based study with all data being recorded on site through the *in-situ* identification and counting of invertebrates. None of the study sites is privately-owned or protected. We did not remove or damage any of the studied organisms beyond taking one or two specimens or fragments from each species during the early years to confirm species identity. The invertebrates were counted and only small samples were taken for confirming field identification of any new or uncertain individuals, so our methods represent no threat to the species we assessed and none of the species is currently endangered.

Data were collected on the color, shape and size of each species in the field and photographs were taken. The identity of each species was confirmed in the laboratory through morphological and histological examination, based on authoritative keys and texts. Where necessary samples of sponges, hydroids, bryozoans, compound ascidians and other small organisms were brought to the university laboratories and observed using a scanning electronic microscope (SEM) Zeiss (DSM 940A).

### Data analysis

The invertebrate density data are expressed as mean ± standard error (SE). We performed a non-metric multidimensional scaling (NMDS) on a Bray-Curtis dissimilarity matrix for each invertebrate group (Porifera, Cnidaria, Mollusca, Bryozoa, Echinodermata and Ascidiacea). Abundance data were log (x+1) transformed and standardised by sample totals. The results were visualised with a 2D ordination diagram with 95% confidence ellipses around the multivariate centroid of samples from each habitat type. We further used permutational multivariate analysis of variance (PERMANOVA) to test the hypothesis of no significant differences in invertebrate density between reefs (Praia do Forte, Itacimirim, Guarajuba and Abai) and years (3 levels; before, during and after 1997–8 ENSO event). PERMANOVA allows multivariate information to be partitioned according to the full experimental design. It makes no assumptions regarding the distributions of the original variables. All P-values are obtained by permutation tests. All tests were carried out using the type III sum of squares and 4999 permutations under the reduced model [27], [28]. Given the high number of permutations, additional Monte Carlo tests were not necessary to reinforce the permutation P-values obtained [29].

We used the SIMPER procedure (similarity percentages) to exam the contribution of species to dissimilarities between the groupings observed in the ordination analyses. Finally, to investigate the relationship between the measured environmental variables (before, during and after 1997–8 ENSO event) and invertebrate assemblage data the BIOENV routine (Spearman rank correlation method) was used with biological and environmental data collected during each sampling year. This method was used as an exploratory tool and is analogous to multiple regression [30]. BIOENV correlates the similarity matrix derived for the invertebrate assemblages with an equivalent matrix for the suite of environmental measurements collected at each site at each time interval. Results are expressed as a Spearmans correlation coefficient (r), ranked in the order that each single variable or combination of variables best explains the observed assemblage patterns [30]. The results (that can have a maximum value of 1) indicate the proportion of variance in the community data explained by these environmental variables (see [30] for full details). All these analyses were performed with the software package PRIMER (version 6.1.6; PRIMER-E, Plymouth, U.K.) and the PERMANOVA+ module (version 1.0.1. PRIMER-E, Plymouth, U.K.).

## Results

### Environmental data

All environmental variables, except pH and sea surface salinity, were significantly different in 1998 compared with the non-ENSO years (See Table S1 in [15]); there was significantly higher sea surface, seawater, and average air temperatures in 1998. There was lower sky cover and lower turbidity during 1998 compared to other years, which correlated with higher levels of ultraviolet radiation reaching the invertebrates in 1998. This year was also characterized by warmer air and sea temperatures, reduced cloud cover and rainfall, higher incoming solar radiation, and reduced turbidity (mainly due to reduced river runoff following decreased precipitation). There was no significant difference in any of the parameters between the reefs, and within the first 2 years (non-ENSO) there was little variability between months.

### Coral species

**Figure 1:**
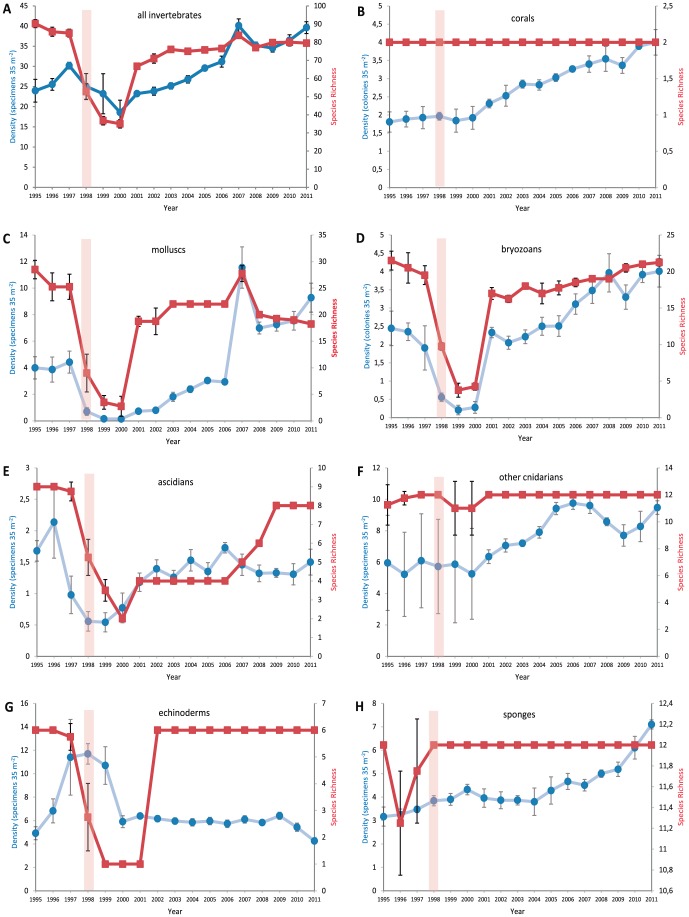
Assemblage metrics from faunal surveys across coastal emergent reefs in Bahia. (A) all invertebrates, (B) corals, (C) molluscs (D) bryozoans, (E) ascidians, (F) cnidarians, (G) echinoderms, (H) sponges. • Density, ▪ Species Richness (additional y-axis). Coloured bars indicate the period of stress.

**Figure 2:**
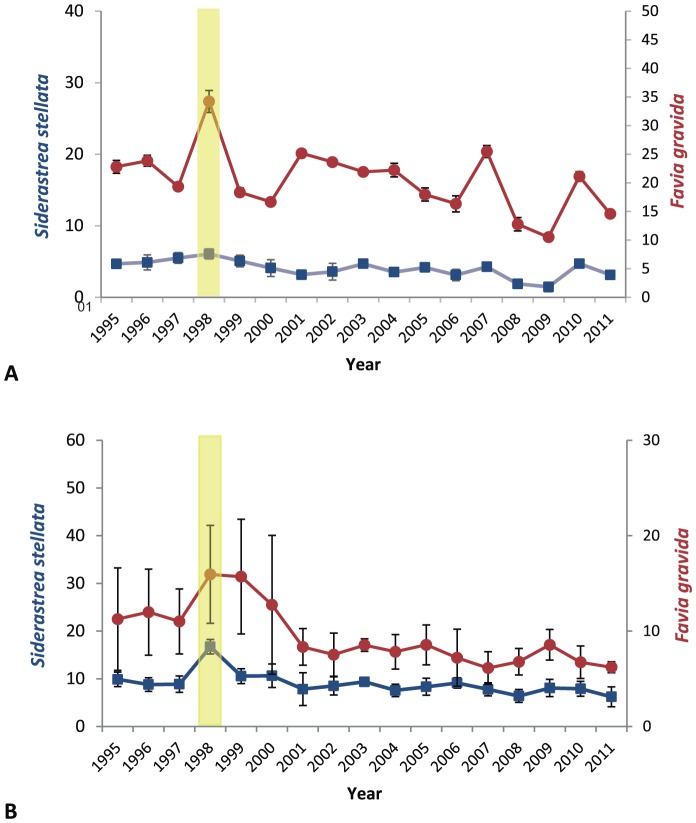
Proportion (%) of coral bleaching and mortality of the scleractinian species from the emergent intertidal reefs of Northern Bahia throughout the 17-year investigation: (A) coral bleaching and (B) coral mortality; (▪) *Siderastrea stellata* and (•) *Favia gravida* (additional y-axis). Coloured bars indicate the period of stress.

The reef tops were colonised by two endemic coral species, *Favia gravida* and *Siderastrea stellata*. Despite some evidence to support the existence of other species of *Siderastrea* (*S. radians*, *S. siderea* and a third unidentified species) along the coast of Bahia, we are confident that only *S. stellata* occurs on the reefs we studied. These species, each with a small polyp diameter, are able to cope with daily aerial exposure and the associated variation in temperature, sunlight, desiccation, and occasional salinity reductions during heavy rain [6]. The densities of these two species were not significantly different between 1998 and the earlier sampling years; however, significant density increases (PERMANOVA, pseudo-F = 3.124; P-*perm*<0.001) were observed from 2001 until the end of the study (Fig. 1B). Bleached colonies of both species were apparent during the whole investigation but was significantly higher during the 1997–8 ENSO than in previous years (PERMANOVA, pseudo-F = 3.852; P-*perm*<0.001; Fig. 2), reaching 40% of total colonies.

### Associated invertebrate community

In addition to the corals, the associated invertebrate community comprised sponges (12 species), non-coral cnidarians (12 species), bryozoans (25 species), molluscs (34 species), echinoderms (6 species) and ascidians (11 species). The most noteworthy impact of the 1997–8 mortality event on the structure of various reef-associated assemblages was the sudden overall loss of species and subsequent decline in species richness (Fig. 1). For all taxa combined, species richness declined significantly on the reef tops from 1998 (PERMANOVA, pseudo-F = 5.336; P-*perm*<0.002), with no indication of recovery during the subsequent two years (Fig. 1A). However, richness recovered markedly in 2001, although by 2011 the overall number of species still remained lower than before the ENSO event.

Overall, while we recorded significant decreases in the densities of the reef-associated invertebrates (PERMANOVA, pseudo-F = 4.504; P-*perm*<0.001 – see table S1 for complete *post-hoc* PERMANOVA results), some phyla appeared more affected than others or responded at different times to the stress imposed during the ENSO event. Densities of molluscs decreased significantly (Fig. 1C) in the post-ENSO years (PERMANOVA, pseudo-F = 2.288; P-*perm*<0.001) and the lowest mean density (0.15±0.01 ind m^−2^) was recorded in 2000; richness showed a similar pattern (PERMANOVA, pseudo-F = 3.913; P-*perm*<0.001), with no live molluscs found on the top of the Abai reef during 2000 survey. The density of bryozoans (Fig. 1D) declined significantly in 1998 (PERMANOVA, pseudo-F = 3.137; P-*perm*<0.001) and continued to decline until 2000, with recovery evident from 2001. The densities of ascidians (Fig. 1E) dropped significantly in 1998 and again in 1999 (PERMANOVA, pseudo-F = 15.686; P-*perm*<0.001), when the lowest density (0.54±0.01 ind m^−2^) was recorded for this group. The density of non-coral cnidarians (Fig. 1F) did not decline significantly in 1998, but their densities have progressively increased, reaching values by the end of the study significantly above those previously recorded (PERMANOVA, pseudo-F = 4.640; P-*perm*<0.001). Echinoderm density increased from the start of the study until 1998 (Fig. 1G), when the highest abundance was recorded; however, densities significantly reduced between 1998 and 2000 (PERMANOVA, pseudo-F = 3.032; P-perm<0.003); densities then remained relatively constant. By 2011, molluscs, bryozoans and ascidians have not recovered to diversity levels observed at the start of the survey pre-ENSO, whereas the number of echinoderm species recovered from 2002 after a major diversity crash (Fig. 1G); only *Echinometra lucunter* was found on reefs in 1999. Sponges (Fig. 1H) did not show any change in density as a result of the ENSO, and they were one of the few groups that became more abundant post-ENSO, compared with pre-ENSO years.

**Figure 3:**
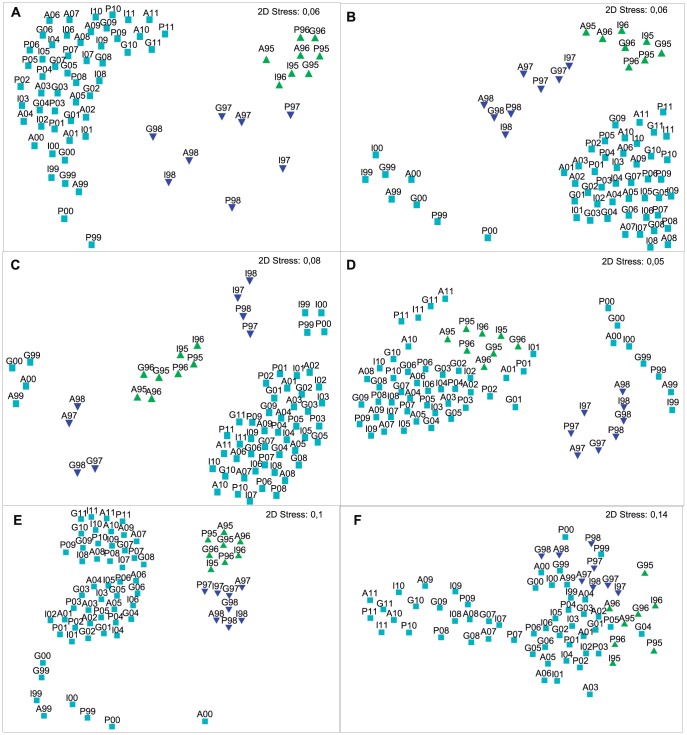
MDS ordinations of the reef-associated invertebrate community data from the emergent intertidal reefs from northern Bahia throughout the sampling period, 1995–2011, based on [ln (x + 1)] transformed species densities and Bray Curtis similarities. (A) ascidians; (B) bryozoans; (C) cnidarians; (D) echinoderms; (E) molluscs; (F) sponges.

**Figure 4:**
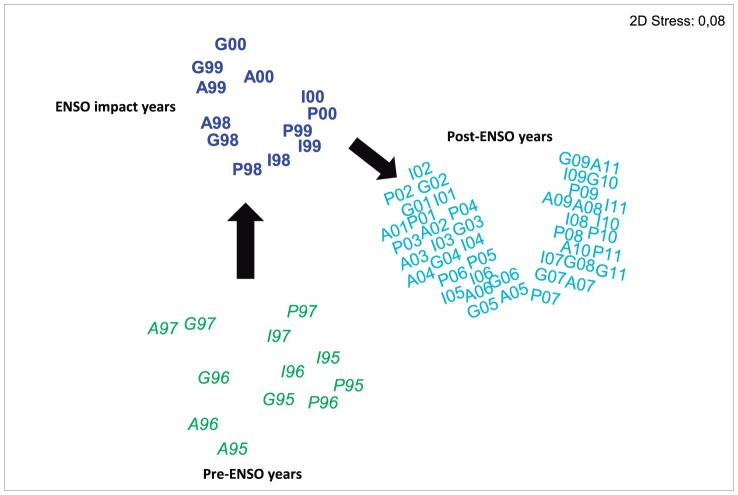
MDS ordination of the reef-associated invertebrate assemblage data from the emergent intertidal reefs in northern Bahia throughout the sampling period, 1995–2011, based on [ln (x + 1)] transformed species densities and Bray Curtis similarities (Average dissimilarity: pre-ENSO×ENSO = 21.86; pre-ENSO×Post-ENSO = 21.24; ENSO×Post-ENSO = 28.18). Arrows indicate the direction of change.

Multivariate analysis of the assemblage composition for each phylum (Fig. 3A–F) clearly demonstrates that the invertebrate community suffered intense modifications throughout the 17-year study, experiencing a dramatic change following the 1997–8 ENSO event. Whilst the community appears to have stabilized since 2001, with an overall greater similarity of all samples taken since this year compared with previous years, it is clear that the new stable assemblage is different from that found prior to 1998, indicating a different rather than a fully recovered community (Fig. 4).

The similarity percentages procedure (SIMPER) indicated that *Stenoplax purpurascens* (1.98), *Lissoclinum perforatum* (1,64), *Fissurella nimbosa* (1.29), *Pseudoactinia melanaster* (1.11) and *Tridentata marginata* (0.96) contributed most to the dissimilarities between pre- and post-ENSO years (Table S2). A number of previously ubiquitous species of molluscs, bryozoans, echinoderms and ascidians disappeared from the reef environment during the ENSO period [16], [17], [31], [32]. Several of these had still not returned 13 years after the impact including: ascidians - *Echinoclinum verrilli*, *Clavelina oblonga*, *Phallusia nigra* and *Botryllus schlosseri*; bryozoans - *Buskia repens*, *Cupuladria canariensis* and *Discoporella buski*; echinoderms - *Ophioderma cinereum* and *Ophiocoma wendtii*; and particularly molluscs - *Ischnochiton dorsuosus*, *Ischnochiton erythronotus*, *Ischnochiton Ischnochiton edwini*, *Ischnochiton Ischnochiton pectinatus*, *Diodora listeria*, *Fissurella nimbosa*, *Fissurella clenchi*, *Cymatium corrugatum*, *Coralliophila aberrans*, *Coralliophila caribaea*, *Leucozonia ocellata* and *Bullata bullata.*

High levels of mortality were observed for several prominent space occupiers and grazers (polyplacophorans, archaeogastropods and gastropods), which was followed by increased densities of clioniid sponges (*Cliona celata* complex and *C. delitrix*), the echinoid *Echinometra lucunter* and the boring ascidian *Lissoclinum perforatum*.

**Figure 5:**
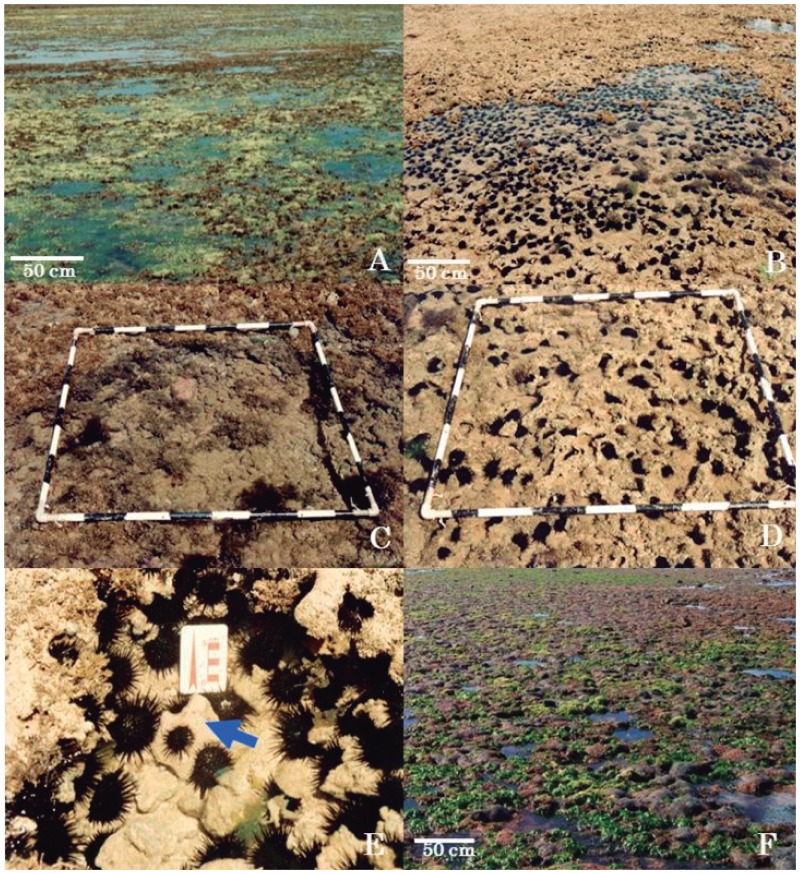
Opportunistic behaviour of *Echinometra lucunter* on the emergent intertidal reefs from northern Bahia after the 1997–8 ENSO event. (A) The reef top of Itacimirim before the ENSO event (April 1996). (B) The same reef area one year after the end of the stress period (April 1999). (C) A quadrat showing the coastal emergent reef top of Praia do Forte before the ENSO event (April 1996). (D) A quadrat of the reef top of Praia do Forte one year after the end of ENSO event (April 1999). (E) Colonies of *Siderastrea stellata* being attacked by echinoids grazing on the top of Guarajuba reef in April 1999. (F) The reef top of Praia do Forte in April 2011. Quadrats: 1 m^2^.

A dramatic change in the reefscape, from a predominantly live reef in 1996 to a sea urchin dominated community had occurred by 1999 (Fig. 5). However, the increased densities of sea urchins did not occur over the entire reef substratum; they were patchily distributed. Echinoid densities slowly decreased to pre-ENSO levels from 2000, but the reef had suffered major bioerosion during the years of elevated grazing pressure (Fig. 5).

The highest correlation identified using the BIOENV analysis on the reef of interest was found for salinity, sunlight irradiation and seawater temperature (r = 0.442).

## Discussion

There is considerable interest in the impacts of global climate change and extreme climatic events on coral reef organisms [4]. Here we documented the impacts of the 1997–8 ENSO event on emergent intertidal systems. Given these communities are regularly exposed at low tide and experience extremes of temperature, we hypothesised that they would be relatively unaffected by the 1997–8 ENSO event. Despite this expectation, the reef-associated populations on Bahia's emergent intertidal systems were dramatically impacted; however, this disturbance had a differential effect on components of the examined communities. The highest mortalities post-ENSO were suffered by the mobile organisms (molluscs and echinoderms), whilst coral, sponges and bioeroders were little affected or showed increases in abundance. We also observed that species that were previously unrecorded prior to the ENSO event (e.g. *Didemnum granulatum*, *D. perlucidum*, *Aplidium lobatum*, *Stenoplax purpurascens* and *Smittipora tuberculata*) are now part of the reef associated biota.

Given that sponges are suspension feeders, we might have expected that a decline in plankton production, which has been previously documented for ENSO events [10], [11], might disrupt sponge trophic links resulting in a decline in the sponge populations; however, this was not observed, even though functionally similar taxa such as bryozoans and ascidians were severely affected. This suggests that sponges have some differential ability to deal with increased temperature (and other stress resulting from the ENSO event) compared to these other groups and may potentially use other food resources compared with bryozoans and ascidians. Recently, there has been increased interest in sponges as there is mounting evidence that they may be tolerant to increased sea surface temperature and ocean acidification, and that coral reefs might be increasingly dominated by sponges [33]. Our results provide further support for this hypothesis and demonstrate the resilience of sponges to climatic anomalies and is consistent with results found for subtidal communities in the same area [34].

Most ascidian species were affected by the ENSO conditions [32], however, the boring species *Lissoclinum perforatum* increased its density throughout the post-ENSO period. We propose that this is due to its ability to reproduce both sexually and asexually [35] and therefore take advantage of the newly available space once other species had declined. This species also possesses chemical defenses against predators [36], [37], which supports its domination of the reef tops when other food resources for predators and grazers are declining.

In contrast to sponges and corals, the decrease in density of molluscs was accompanied by a reduction in diversity as species became progressively less abundant after the 1997–8 ENSO event. Molluscs have the capacity to migrate to sheltered subtidal zones during the thermal stress and avoid prolonged exposure to excessive heat. However, the species that declined were not recorded within other reef habitats such as reef walls and shallow banks [17], [32], suggesting migration from the intertidal was not a viable strategy available to these species.

The severe impact on the echinoderm community was particularly surprising. All six species recorded are regarded as relatively resistant to extremes of temperature as they are exposed on a daily basis during low tide to variation in temperature, solar radiation exposure, desiccation and salinity oscillations (especially during the rainy season; see [6]). However, all but *E. lucunter* disappeared from the reef top fauna following the ENSO event and were not seen again until 2002. It is therefore possible that these species (as well the other invertebrates that declined in abundance) are at the very edge of their range of physiological tolerance to temperature within these intertidal reef systems and the severe conditions recorded during the ENSO period proved too great a stress. In addition, the reef top echinoderm assemblage appeared to be much more dramatically impacted than those from the subtidal reef walls and shallow banks (see [38]).

In 1995, when the monitoring program began, a rich community (95 species) was present in the northern Bahian intertidal reefs; however, in 2000, this was reduced to only 39 species, mostly sponges and cnidarians (12 each). By the end of the survey 82 species inhabited the studied area, equating to a diversity still 13.7% lower than before the ENSO event. This does, however, indicate that diversity can recover following such high temperature events. The few colonies of *S. stelatta* that suffered partial mortality or died in 1997–8 had been subject to intense bioerosion (Figure 5E), although the densities of the opportunistic echinoids partly causing this bioerosion has reduced since 2000; however, they are still more abundant than recorded in 1995.

While coral bleaching in response to changes in global climate is a major concern worldwide, not all corals respond to temperature stress in the same way. In an earlier study, Jokiel and Coles [39] showed that individual coral colonies living in high temperature environments can survive and their symbionts photosynthesise at temperatures at several degrees higher than their congeners living at lower temperature environments [40], [41]. Depending upon the location, these species were able to withstand sustained temperatures of 30°C for several weeks [42], [43] and 32–34°C from several days to a few weeks [44]. Clausen and Roth [45] suggested that many species are able to physiologically acclimatise to increased temperatures, which has been validated by a number of more recent studies (e.g. [46], [47]) and by the response displayed by *F. gravida* and *S. stellata* in our study. These species were resistant to the elevated temperatures recorded in northern Bahia during the ENSO period. In fact densities of both these species increased over the study period and significant change was noticed neither between 2001 and 2005, when further thermal anomalies varying from 0.25°C to 0.75°C were reported for the coast of Bahia [48], nor during the 2009–10 ENSO that caused bleaching in other regions [49]. This reinforces the hypothesis of heat-adaptability [47], [50] and that the studied corals are locally adapted to temperature fluctuations [6], [16] and should be considered as models for further study of local temperature tolerance in corals.

The recovery of the organisms that suffered partial mortality in 1997–8 was further inhibited by the delayed effects of the intense solar radiation in the study area [15], [16]. For example, this may have compromised the sexual reproduction and larval settlement success in future seasons [51], [52], and may explain the absence of coral recruits during 1999–2000. Therefore, initial recolonisation of northern Bahian emergent reefs was result of new-born colonies. Juvenile colonies, particularly of the endemic *Siderastrea stellata*, were apparent for the first time in May 2001 [14]. Similarly, there was a marked recovery in the diversity and density of the invertebrate fauna from 2001 (Fig. 1A) after a continued decrease in 1997–2000. Despite the dramatic impact on the Bahian reef fauna, there was, therefore, no evidence of a longer-term shift to a macroalgal dominated state as seen in parts of the Caribbean.

Given that it took 13-years for the reefs we studied to recover to a stable community from the 1997–8 ENSO event, it is reasonable to assume that if ENSO events occur more regularly with periods of persistent sea warming, then surviving coral populations, reef associated invertebrates and framework structures would be subject to increasing levels of predation and bioerosion. We propose that if the frequency of extreme ENSO events increases, then the recovery capacity for coral reef assemblages will be progressively diminished with each ENSO event. Given that ENSO phenomena are cyclical and that strong events can generate extreme disturbance resulting in the mortality of both symbiotic and non-symbiotic reef organisms, if global sea temperatures increase to levels comparable to the 1997–8 event, mortality of corals and other reef associated organisms is more likely as species will be even closer to their thermal maxima. In addition, from our results there are also likely to be increases in the abundance of predators and bioeroders. This would further increase reef-mortality and bioerosion, potentially leading to a rapid destruction of the reef framework, and a reduced capacity for subsequent recovery.

In conclusion, we found differential effects of the 1997–8 ENSO event on the fauna of the emergent reefs in northern Bahia. Corals and sponges appeared to be particularly resilient. Importantly, our study demonstrates that even 13-years after the event, the communities had not returned to their original state and overall diversity was lower. It is unclear if the community will continue to recover given more time, but our study highlights that any increase in the frequency of large-scale climatic events to more than one a decade is likely to result a persistent lowered diversity state. Finally, we showed that the corals and sponges in these environments appeared relatively unimpacted by the ENSO event, and therefore represent future models for understanding the potential resilience of marine organisms to climate change.

## Supporting Information

Table S1 **Similarity percentages analysis (SIMPER): Species of reef associated invertebrates contributing most to the dissimilarity between pre-ENSO and post-ENSO years.** Average dissimilarity = 38,01.
(DOC)

Table S2 **Post-Hoc results for differences in reef-associated assemblages between Reefs/Years measured from 1995 to 2011 tested by a distance-based permutational multivariate analysis of variance, PERMANOVA.** Note: 4999 permutations; transformation log(x+1); Bray Curtis dissimilarity. Contrasts degrees of freedom: pre-ENSO×ENSO = 3, pre-ENSO×post-ENSO = 13, ENSO×post-ENSO = 13.
(DOC)
