# Supplementary material for: Differential Responses of Emergent Intertidal Coral Reef Fauna to a Large-Scale El-Niño Southern Oscillation Event: Sponge and Coral Resilience
Source: PLoS One. 2014 Mar 27;9(3):e93209. doi: 10.1371/journal.pone.0093209 (PMC3968116; doi:10.1371/journal.pone.0093209)
Supplement: Table S1 — Similarity percentages analysis (SIMPER): Species of reef associated invertebrates contributing most to the dissimilarity between pre-ENSO and post-ENSO years. Average dissimilarity = 38,01. (DOC) [file pone.0093209.s001.doc]

| Species | pre-ENSO Average Abundance | post-ENSO Average Abundance | Average  Dissimilarity |
| --- | --- | --- | --- |
| *Stenoplax purpurascens* | 0,00 | 0,80 | 1,98 |
| *Lissoclinum perforatum* | 0,11 | 0,71 | 1,64 |
| *Fissurella nimbosa* | 0,47 | 0,00 | 1,29 |
| *Pseudoactinia malanaster* | 0,37 | 0,68 | 1,11 |
| *Tridentata marginata* | 0,55 | 0,36 | 0,96 |
| *Palythoa variabilis* | 0,29 | 0,60 | 0,90 |
| *Discoporella umbellata* | 0,35 | 0,03 | 0,89 |
| *Diodora cayenensis* | 0,37 | 0,06 | 0,87 |
| *Phyllactis praetexta* | 0,42 | 0,69 | 0,83 |
| *Utinga castanea* | 0,08 | 0,37 | 0,77 |
| *Herdmania momus* | 0,29 | 0,02 | 0,77 |
| *Metrarabdotos tenue* | 0,09 | 0,37 | 0,76 |
| *Siderastrea stellata* | 0,78 | 1,06 | 0,75 |
| *Echinometra lucunter* | 1,76 | 1,72 | 0,75 |
| *Bullata bullata* | 0,26 | 0,00 | 0,73 |
| *Clavelina oblonga* | 0,26 | 0,00 | 0,72 |
| *Phallusia nigra* | 0,25 | 0,00 | 0,70 |
| *Cupuladria canariensis* | 0,25 | 0,00 | 0,70 |
| *Echinochinum verrilli* | 0,23 | 0,00 | 0,65 |
| *Bunodosoma cangicum* | 0,61 | 0,72 | 0,65 |
| *Thais rustica* | 0,28 | 0,06 | 0,63 |
| *Siphonodictyon coralliphagum* | 0,14 | 0,36 | 0,63 |
| *Eudendrium carneum* | 0,24 | 0,42 | 0,61 |
| *Favia gravida* | 0,51 | 0,71 | 0,60 |
| *Reptadeonella violacea* | 0,06 | 0,28 | 0,59 |
| *Fissurella clenchi* | 0,21 | 0,00 | 0,59 |
| *Linckia guildinki* | 0,28 | 0,37 | 0,56 |
| *Ophioderma cinereum* | 0,21 | 0,32 | 0,55 |
| *Exechonella brasiliensis* | 0,08 | 0,26 | 0,54 |
| *Tridentata distans* | 0,22 | 0,36 | 0,54 |
| *Diodora dysoni* | 0,25 | 0,07 | 0,53 |
| *Siphonodictyon sp* | 0,06 | 0,25 | 0,48 |
| *Bullata lilacina* | 0,22 | 0,07 | 0,46 |
| *Thyroscyphus ramosus* | 0,69 | 0,83 | 0,45 |
| *Palythoa sp.* | 0,15 | 0,29 | 0,42 |
| *Macrorhynchia phillipina* | 0,18 | 0,13 | 0,41 |
| *Echinaster echinophorea* | 0,18 | 0,26 | 0,41 |
| *Cinachyrella apion* | 0,57 | 0,72 | 0,41 |
| *Cinachyrella alloclada* | 0,61 | 0,73 | 0,41 |
| *Botryllus nigrum* | 0,20 | 0,06 | 0,40 |
| *Styella plicata* | 0,20 | 0,07 | 0,39 |
| *Canda retiformis* | 0,15 | 0,07 | 0,33 |
| *Botryllus schlosseri* | 0,12 | 0,00 | 0,33 |
| *Zoanthus sociatus* | 0,30 | 0,38 | 0,33 |
| *Buskia repens* | 0,12 | 0,00 | 0,32 |
| *Cliona celata* (complex) | 0,68 | 0,74 | 0,30 |
| *Bugula uniserialis* | 0,12 | 0,01 | 0,30 |
| *Thais deltoidea* | 0,14 | 0,06 | 0,27 |
| *Ischnochiton Ischnochiton edwini* | 0,09 | 0,04 | 0,26 |
| *Halocordyle disticha* | 0,13 | 0,13 | 0,22 |
| *Bugula carvalhoi* | 0,12 | 0,09 | 0,22 |
| *Diodora listeri* | 0,08 | 0,00 | 0,22 |
| *Lythechinus variegatus* | 0,09 | 0,09 | 0,19 |
| *Ischnochiton erythronotus* | 0,09 | 0,04 | 0,18 |
| *Ophiocoma wendtii* | 0,07 | 0,10 | 0,18 |
| *Natica pusilla* | 0,08 | 0,06 | 0,18 |
| *Antropora typica* | 0,10 | 0,06 | 0,18 |
| *Serpulobis decussatus* | 0,04 | 0,07 | 0,18 |
| *Cymatium cynocephalum* | 0,10 | 0,06 | 0,18 |
| *Calliostoma bullisi* | 0,07 | 0,07 | 0,16 |
| *Aptonella violacea* | 0,10 | 0,07 | 0,16 |
| *Thais haemastoma* | 0,03 | 0,07 | 0,16 |
| *Astraea tecta olfersii* | 0,02 | 0,07 | 0,16 |
| *Cypraea zebra* | 0,06 | 0,07 | 0,15 |
| *Electra pilosa* | 0,05 | 0,09 | 0,15 |
| *Celleporaria carvalhoi* | 0,07 | 0,06 | 0,15 |
| *Schizoporella carvalhoi* | 0,07 | 0,07 | 0,15 |
| *Cymatium corrugatum* | 0,05 | 0,00 | 0,14 |
| *Collisella marcusi* | 0,02 | 0,06 | 0,14 |
| *Ischnochiton Ischnochiton pectinatus* | 0,07 | 0,04 | 0,14 |
| *Astraea latispina* | 0,05 | 0,07 | 0,13 |
| *Nellia oculata* | 0,05 | 0,09 | 0,13 |
| *Celleporaria shubarti* | 0,07 | 0,06 | 0,13 |
| *Mycale sp2* | 0,02 | 0,07 | 0,13 |
| *Ischnochiton dorsuosus* | 0,05 | 0,04 | 0,13 |
| *Collisella abrolhosensis* | 0,05 | 0,03 | 0,13 |
| *Lucapina philippiana* | 0,05 | 0,06 | 0,12 |
| *Hippothoa flagellum* | 0,07 | 0,05 | 0,12 |
| *Chaetopleura sowerbiana* | 0,04 | 0,04 | 0,11 |
| *Fissurella rosea* | 0,04 | 0,07 | 0,11 |
| *Spirastrella hartmani* | 0,04 | 0,08 | 0,11 |
| *Catenicella contei* | 0,03 | 0,04 | 0,11 |
| *Electra belula* | 0,06 | 0,06 | 0,11 |
| *Perotrochus amabilis* | 0,06 | 0,06 | 0,10 |
| *Desmapsamma anchorata* | 0,05 | 0,08 | 0,10 |
| *Terebripora fisheri* | 0,02 | 0,04 | 0,10 |
| *Spirastrella cunctatrix* | 0,07 | 0,08 | 0,10 |
| *Discoporella buski* | 0,04 | 0,00 | 0,10 |
| *Smittipora tuberculata* | 0,00 | 0,04 | 0,09 |
| *Collisella subrugosa* | 0,03 | 0,00 | 0,09 |
| *Coralliophila aberrans* | 0,03 | 0,00 | 0,08 |
| *Amphimedon viridis* | 0,05 | 0,06 | 0,08 |
| *Anthosigmella varians* | 0,07 | 0,08 | 0,08 |
| *Cribilaria floridana* | 0,03 | 0,04 | 0,08 |
| *Calliotropis calatha* | 0,02 | 0,00 | 0,07 |
| *Chondrilla nucula* | 0,07 | 0,08 | 0,07 |
| *Aplidium lobatum* | 0,00 | 0,03 | 0,06 |
| *Didemnum granulatum* | 0,00 | 0,02 | 0,05 |
| *Didemnum perlucidum* | 0,00 | 0,02 | 0,05 |
| *Membranipora tuberculata* | 0,01 | 0,00 | 0,03 |
| *Leucozonia ocellata* | 0,01 | 0,00 | 0,03 |
| *Coralliophila caribaea* | 0,01 | 0,00 | 0,02 |
