# Supplementary material for: Differential Responses of Emergent Intertidal Coral Reef Fauna to a Large-Scale El-Niño Southern Oscillation Event: Sponge and Coral Resilience
Source: PLoS One. 2014 Mar 27;9(3):e93209. doi: 10.1371/journal.pone.0093209 (PMC3968116; doi:10.1371/journal.pone.0093209)
Supplement: Table S2 — Post-Hoc results for differences in reef-associated assemblages between Reefs/Years measured from 1995 to 2011 tested by a distance-based permutational multivariate analysis of variance, PERMANOVA. Note: 4999 permutations; transformation log(x+1); Bray Curtis dissimilarity. Contrasts degrees of freedom: pre-ENSO×ENSO = 3, pre-ENSO×post-ENSO = 13, ENSO×post-ENSO = 13. (DOC) [file pone.0093209.s002.doc]

|  |  |  | | Reef level | | | | | | | |
| --- | --- | --- | --- | --- | --- | --- | --- | --- | --- | --- | --- |
|  |  |  | Praia do Forte | | | Itacimirim | | Guarajuba | | Abaí | |
|  | Group contrasts | Unique perms | t | | P(*perm*) | t | P(*perm*) | t | P(*perm*) | t | P(*perm*) |
|  |  |  |  | |  |  |  |  |  |  |  |
| Porifera | pre-ENSO x ENSO | 3 | 1.755 | | 0.331 | 1.524 | 0.337 | 1.279 | 0.339 | 1.215 | 0.342 |
|  | pre ENSO x post ENSO | 105 | 1.409 | | 0.073 | 1.689 | 0.055 | 1.806 | 0.061 | 1.366 | 0.114 |
|  | ENSO x post ENSO | 105 | 1.360 | | 0.105 | 1.528 | 0.092 | 1.180 | 0.225 | 1.146 | 0.217 |
|  |  |  |  | |  |  |  |  |  |  |  |
| Cnidaria | pre-ENSO x ENSO | 3 | 1.356 | | 0.334 | 1.363 | 0.329 | 1.499 | 0.332 | 2.236 | 0.331 |
|  | pre ENSO x post ENSO | 105 | 1.967 | | ***0.046*** | 1.692 | ***0.046*** | 1.434 | ***0.049*** | 3.476 | ***0.009*** |
|  | ENSO x post ENSO | 105 | 1.985 | | ***0.018*** | 1.982 | ***0.035*** | 1.708 | ***0.047*** | 2.697 | ***0.019*** |
|  |  |  |  | |  |  |  |  |  |  |  |
| Bryozoa | pre-ENSO x ENSO | 3 | 1.149 | | 0.337 | 1.174 | 0.336 | 1.299 | 0.331 | 1.353 | 0.338 |
|  | pre ENSO x post ENSO | 105 | 2.004 | | ***0.020*** | 2.011 | ***0.021*** | 1.801 | ***0.028*** | 2.097 | ***0.022*** |
|  | ENSO x post ENSO | 105 | 1.824 | | ***0.048*** | 1.467 | ***0.053*** | 1.635 | ***0.049*** | 1.815 | ***0.044*** |
|  |  |  |  | |  |  |  |  |  |  |  |
| Mollusca | pre-ENSO x ENSO | 3 | 0.959 | | 0.661 | 0.957 | 1.000 | 0.738 | 1.000 | 0.867 | 1.000 |
|  | pre ENSO x post ENSO | 105 | 1.554 | | ***0.042*** | 1.579 | ***0.034*** | 1.872 | ***0.015*** | 1.680 | ***0.016*** |
|  | ENSO x post ENSO | 105 | 1.282 | | ***0.011*** | 1.597 | ***0.024*** | 1.607 | ***0.017*** | 1.378 | ***0.047*** |
|  |  |  |  | |  |  |  |  |  |  |  |
| Echinoderma | pre-ENSO x ENSO | 3 | 2.942 | | 0,328 | 1.946 | 0.329 | 2.326 | 0.334 | 3.334 | 0.337 |
|  | pre ENSO x post ENSO | 105 | 0.543 | | 0.665 | 0.485 | 0.785 | 1,214 | 0.248 | 0.565 | 0.752 |
|  | ENSO x post ENSO | 105 | 2.065 | | ***0.047*** | 2.064 | ***0.044*** | 2.755 | ***0.018*** | 2.340 | ***0.051*** |
|  |  |  |  | |  |  |  |  |  |  |  |
| Ascidiacea | pre-ENSO x ENSO | 3 | 1.148 | | 0.337 | 1.288 | 0.330 | 2.251 | 0.322 | 1.916 | 0.339 |
|  | pre ENSO x post ENSO | 105 | 4.590 | | ***0.010*** | 6.166 | ***0.012*** | 6.166 | ***0.008*** | 5.519 | ***0.010*** |
|  | ENSO x post ENSO | 105 | 3.074 | | ***0.010*** | 3.416 | ***0.011*** | 2.537 | ***0.009*** | 3.516 | ***0.010*** |
|  |  |  |  | |  |  |  |  |  |  |  |
| All invertebrates | pre-ENSO x ENSO | 3 | 1.515 | | 0.336 | 1.384 | 0.328 | 1.497 | 0.335 | 1.646 | 0.326 |
|  | pre ENSO x post ENSO | 105 | 2.126 | | ***0.019*** | 2.177 | ***0.020*** | 2.312 | ***0.021*** | 2.689 | ***0.009*** |
|  | ENSO x post ENSO | 105 | 1.871 | | ***0.048*** | 1.961 | ***0.039*** | 1.976 | ***0.061*** | 2.203 | ***0.026*** |
|  |  |  |  | |  |  |  |  |  |  |  |
